# Anaplastic Transformation of Follicular Thyroid Carcinoma in Pulmonary Metastasis With Gradually Progressive Intra-tumoral Cavitation: A Case Report

**DOI:** 10.7759/cureus.31999

**Published:** 2022-11-29

**Authors:** Hajime Torizuka, Minoru Inoue, Yusuke Iizuka, Yosuke Yamada, Takashi Mizowaki

**Affiliations:** 1 Department of Radiation Oncology and Image-applied Therapy, Kyoto University Graduate School of Medicine, Kyoto, JPN; 2 Department of Radiation Oncology, Shizuoka City Shizuoka Hospital, Shizuoka, JPN; 3 Department of Diagnostic Pathology, Kyoto University Hospital, Kyoto, JPN

**Keywords:** differentiated thyroid carcinoma, anaplastic thyroid cancer, pulmonary metastases, tumor cavitation, i-131 radioiodine treatment

## Abstract

Anaplastic transformation of differentiated thyroid cancer is rare but clinically important because of the dismal prognosis after anaplastic transformation. Therefore, cases and findings of anaplastic transformation must be accumulated, which could ultimately lead to an earlier diagnosis and an improved prognosis. Here, we present a case of anaplastic transformation of follicular thyroid carcinoma (FTC) in a pulmonary metastatic lesion associated with gradually progressive tumor cavitation. The patient with FTC was diagnosed with multiple lung metastases three years after surgery for the primary tumor and metastatic neck lymph nodes. Annual treatment with radioactive iodine resulted in disease stability for 10 years. However, one lung metastasis in the left lower lobe gradually enlarged and was associated with intra-tumoral cavitation. The growing lung nodule was resected and pathologically diagnosed as an anaplastic transformation of FTC. Fourteen months after diagnosis, the patient died of pneumothorax caused by pleural dissemination despite multiple treatment interventions. This case highlights pulmonary metastasis with progressive cavitary lesions as a potential early sign of the anaplastic transformation of differentiated thyroid cancer.

## Introduction

The incidence of thyroid cancer has increased worldwide over the past three decades [1]. Most cases are differentiated thyroid carcinomas (papillary or follicular thyroid carcinoma, (PTC or FTC)), which are routinely managed surgically with or without radioactive iodine (RAI) therapy. In general, PTC and FTC are biologically indolent with excellent prognoses, with 10-year survival rates exceeding 80% [2]. However, a small subset of PTC and FTC cases transform into anaplastic carcinoma. Anaplastic transformation occurs within primary tumors [3], metastatic lymph nodes [4], and metastatic lesions in distant organs [5]. Patients with anaplastic transformation have a poor prognosis, with six-month survival rates of only 35% [6]. Therefore, it is essential to accumulate further cases of anaplastic transformation in PTC and FTC, which could lead to the identification of predictive imaging features and biomarkers for detecting patients with an elevated risk of anaplastic transformation. Herein, we report a case of anaplastic transformation of FTC arising from a lung metastasis that exhibited gradually progressive intra-tumoral cavitation.

## Case presentation

**Figure 1:**
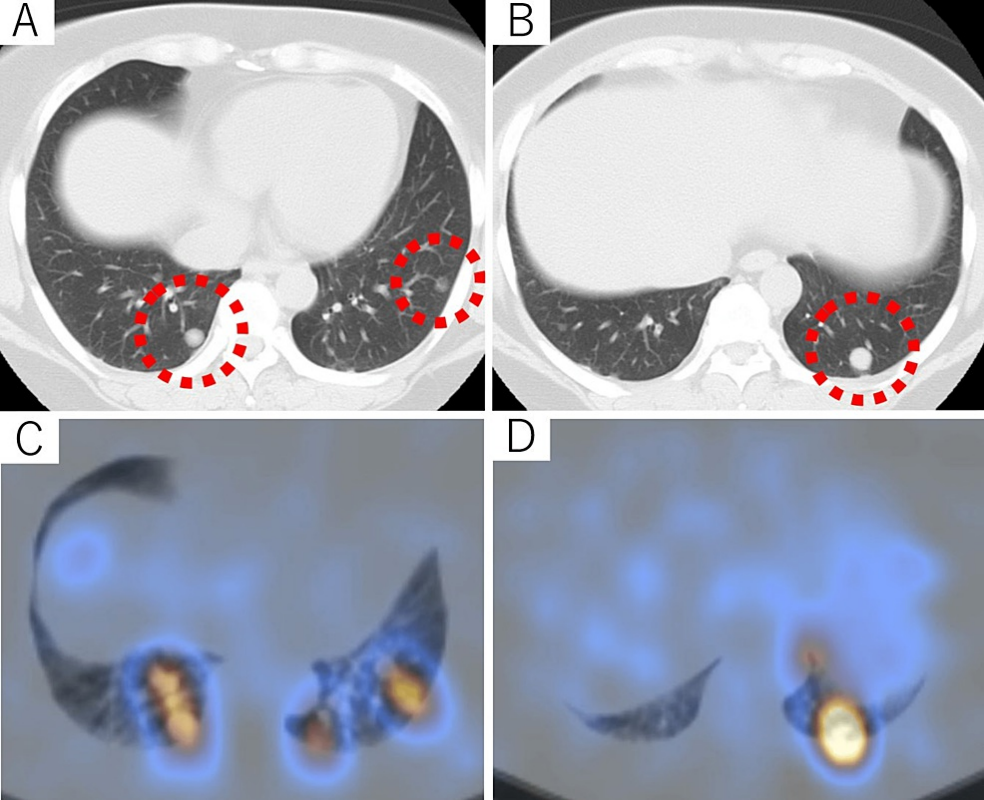
Representative images of the multiple pulmonary metastases (A, B) A chest CT scan performed at the time of the initial diagnosis of pulmonary metastases. (C, D) Scintigraphy with SPECT/CT performed five days after 131I administration. CT: computed tomography; SPECT: single photon emission computed tomography; 131I: iodine-131.

A 33-year-old woman had been aware of her slowly growing thyroid gland since 1997. In 2002, she recognized a palpable mass in the right thyroid, which led to the detection of biopsy-proven thyroid cancer. Right thyroid lobectomy and cervical lymph node dissection were performed in the same year. Postoperative pathological examination revealed FTC of the primary tumor and metastatic cervical lymph nodes (pT3N1bM0, pStage I (UICC 8th edition)). An additional total thyroidectomy was not performed as per the patient’s request. In 2005, multiple asymptomatic lung metastases were identified on thoracic computed tomography (Figures 1A, 1B). Therefore, the patient underwent residual thyroidectomy and subsequent RAI therapy (iodine-131 (131I): 4.81 GBq) in 2006. An uptake of 131I was observed in the thyroid bed and lung metastases (Figures 1C, 1D).

**Figure 2:**
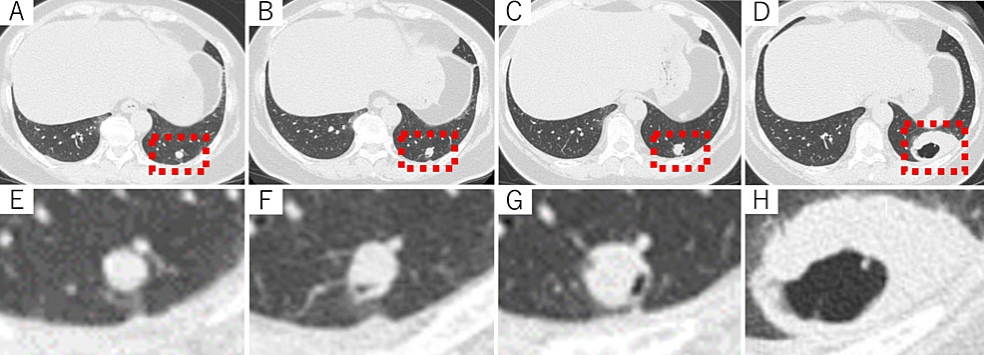
Pulmonary metastasis associated with gradually progressive intra-tumoral cavitation and subsequent anaplastic transformation (A-D) Chest CT scans taken (A) 10 years, (B) 11 years, (C) 12 years, and (D) 13 years after pulmonary metastasis diagnosis. (E-H) Enlarged views of the pulmonary metastasis are shown in Figures 2A, 2B, 2C, and 2D, respectively.

The patient further underwent nine doses of 131I between 2006 and 2016 (4.81 GBq×3 and 5.55 GBq×6). In each treatment, 131I was well distributed to the lung metastases, and the patient had sustained stable disease (i.e., no remarkable changes in serum thyroglobulin (Tg) concentration or lung metastasis size). However, one metastatic nodule in the left lower lobe displayed a slight increase associated with intra-tumoral cavitation (Figures 2A, 2B, 2E, 2F) and weakened uptake of 131I after the last two RAI therapies. In June 2019, this lung nodule exhibited remarkable growth from 9 mm to 52 mm (Figures 2C, 2D, 2G, 2H), whereas the serum Tg level decreased from 194.6 ng/mL to 98.4 ng/mL.

**Figure 3:**
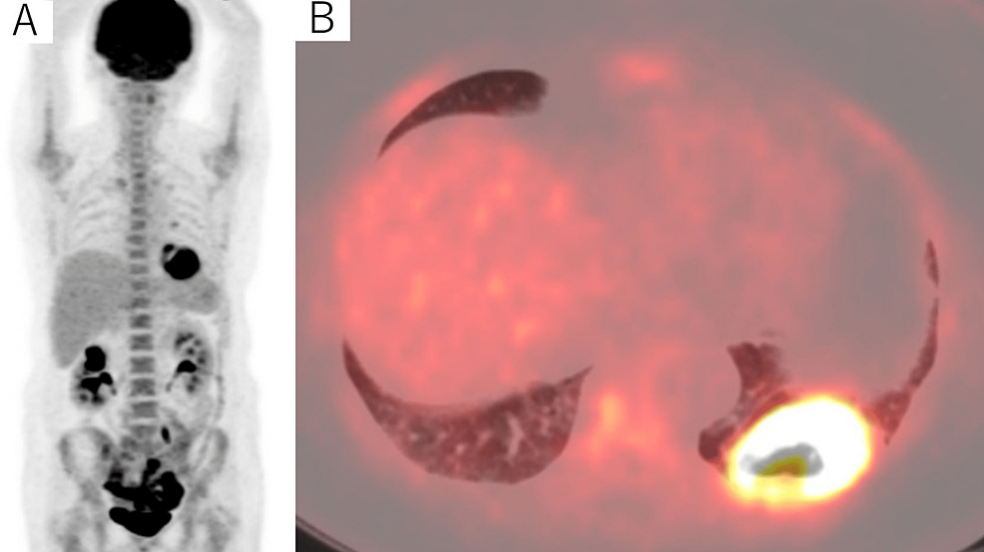
Representative images of FDG PET/CT (A) Maximal intensity projection image of whole-body 18F-FDG uptake; (B) Axial plane of the fusion PET/CT image 18F-FDG: fluorine-18-fluorodeoxyglucose; PET: positron emission tomography

Fluorine-18-fluorodeoxyglucose (^18^F-FDG) positron emission tomography (PET) revealed significant ^18^F-FDG uptake only in this nodule (Figures 3A, 3B), indicating dedifferentiation or anaplastic transformation of the lung metastasis of FTC.

Consequently, in July 2019, a left lower lung lobectomy was performed to establish the diagnosis and determine the optimal treatment strategy.

**Figure 4:**
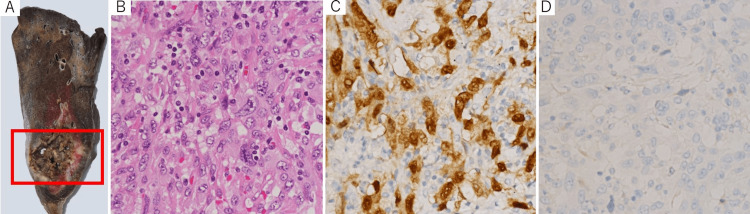
Pathological findings of the resected lung tumor (A) Macroscopic image of the resected lung. The tumor associated with intra-tumoral cavitation is shown in the red square. (B) Hematoxylin and eosin stain. (C, D) Immunohistochemical staining for PAX8 (C) and TTF-1 (D) PAX8: paired-box gene 8; TTF-1: thyroid transcription factor-1.

Histopathological evaluation of the resected lung (Figure 4A) revealed that the enlarged tumor was highly pleomorphic (Figure 4B), paired-box gene 8 (PAX8) positive (Figure 4C), and thyroid transcription factor-1 (TTF-1) negative (Figure 4D), compatible with anaplastic thyroid carcinoma [7].

**Figure 5:**
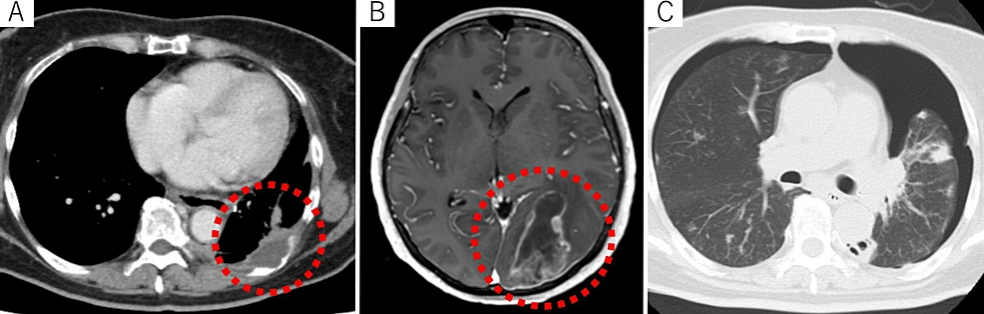
Systemic progression following the diagnosis of anaplastic transformation of follicular thyroid cancer (A) Pleural dissemination with costal bone invasion. (B) Brain metastasis. (C) Pneumothorax due to the progression of pleural invasion.

Other small nodules in the resected lung exhibited follicular differentiation and were PAX8 and TTF-1 positive (data not shown). Thus, anaplastic transformation in the pulmonary metastasis of FTC was diagnosed. Based on this diagnosis, 24 mg/day of lenvatinib was initiated. However, the antitumor effect of lenvatinib was partial (i.e., a mild decrease in serum Tg level). The patient exhibited pleural dissemination in October 2019 (Figure 5A), lymph node metastases in the neck and mediastinum in January 2020, bone metastases in the zygomatic bone in February 2020, and multiple brain metastases in July 2020 (Figure 5B). Although a series of palliative external beam radiotherapy treatments were administered (25 Gy/5 fractions for the pleural dissemination, 17.5 Gy/8 fractions for the lymph nodes in the mediastinum, and 20 Gy/5 fractions of whole brain radiotherapy for multiple brain metastases), sufficient shrinkage of the tumor and palliation of the symptoms were not achieved. Finally, the patient died of respiratory failure due to pleural dissemination-derived pneumothorax in October 2020 (Figure 5C).

## Discussion

Anaplastic thyroid carcinoma (ATC) accounts for 2% of all thyroid cancers and has an extremely poor prognosis, with one-year survival rates of approximately 5% [8]. The majority of ATC cases are considered to be transformed from pre-existing PTC or FTC, whereas some arise de novo from pre-existing multinodular goiters. Various genetic and epigenetic alterations related to anaplastic transformation (such as TERT promoter and TP53) have been identified [4]. However, knowledge of the clinical signs of anaplastic transformation remains limited. Rapid proliferation without elevation of the tumor marker Tg [9], loss of uptake of RAI [10], and newly acquired uptake properties of 18F-FDG [10] are potential signs of anaplastic transformation of PTC and FTC. In addition, the current case exhibited gradually progressive intra-tumoral cavitation before the diagnosis of anaplastic transformation of the FTC.

Several reports have described the formation of intra-tumoral cavitation in ATC. Micro-vessel density in the tumor tissue of ATC is significantly lower than that in the tumor tissue of PTC and FTC, which may contribute to the development of intra-tumoral cavitation through tumor necrosis mediated by a lack of blood supply [11]. Furthermore, pharmacological inhibition of tumor angiogenesis by bevacizumab [12] and lenvatinib [13] also promotes pulmonary tumor cavitation. The increasing demand for blood supply within the tumor undergoing anaplastic transformation may lead to intra-tumoral cavitation, further suggesting that the formation of intra-tumoral cavitation may be an early sign of anaplastic transformation of pulmonary metastases.

The establishment of therapeutic strategies against ATC is warranted because standard treatment approaches for differentiated thyroid cancers (i.e., thyroidectomy and RAI therapy) are mostly ineffective for ATC owing to the disease extent and loss of the ability to uptake iodine [14]. The efficacy of systemic therapy (e.g., paclitaxel with or without carboplatin, doxorubicin with or without docetaxel, sorafenib, pazopanib, and lenvatinib), as well as radiotherapy, is also limited [15-19]. In cases of BRAF V600E-mutant ATC, combined therapy with dabrafenib and trametinib has proven more effective, with a median overall survival of 15 months and a 12-month survival rate of 52% [20]. Unfortunately, the Japanese national health insurance system does not cover the costs of dabrafenib plus trametinib treatment for ATC. Therefore, this regimen was not considered in the present case.

## Conclusions

The presented case demonstrates the anaplastic transformation of FTC in pulmonary metastasis. Additionally, it highlights the development of intra-tumoral cavitation as a potential early sign of anaplastic transformation in the pulmonary metastasis of differentiated thyroid cancer. Although further investigations and the accumulation of similar cases are needed, our findings may stimulate future research exploring the radiologic features of anaplastic transformation and ultimately support clinicians by facilitating an early diagnosis.
